# Different Results from Varied Angles: The Positive Impact of Work Connectivity Behavior After-Hours on Work Engagement

**DOI:** 10.3390/bs13120971

**Published:** 2023-11-25

**Authors:** Yang Yang, Rui Yan, Xuehong Li, Yan Meng, Guoqiang Xie

**Affiliations:** School of Management, Harbin Institute of Technology, Harbin 150006, China

**Keywords:** work connectivity behavior after-hours, work autonomy, emotional exhaustion, work engagement

## Abstract

With the development of communication technology and the COVID-19 pandemic, it has become increasingly common for employees to maintain work connectivity after-hours, which has a significant impact on their psychological state at work. However, most of the existing studies have not reached a consensus on the impact of work connectivity behavior after-hours on employees’ psychological state at work, and the existing studies have led to theoretical and practical disagreements. Based on the Job Demands–Resources model, we built a two-path model of work autonomy and emotional exhaustion to explore the impact of work connectivity behavior after-hours on work engagement. In addition, we compared the differences between different workplace statuses (managers and ordinary employees). Through surveys and analyses of 257 employees, the results show that work connectivity behavior after-hours positively impacts employees’ work engagement by increasing managers’ work autonomy and reducing ordinary employees’ emotional exhaustion. This study not only reveals that work connectivity behavior after-hours positively affects work engagement but also illustrates the differences in impact between managers and ordinary employees; these findings contribute to the development of a consensus on the influence of work connectivity behavior after-hours on employees’ psychological state at work, which provides insights for organizations seeking to manage work connectivity behavior after-hours, for example, by adopting different connectivity management strategies for employees with different workplace statuses.

## 1. Introduction

Information and communication technologies (ICTs) are becoming increasingly important in organizations, especially to mitigate the impact of “black swan” events (e.g., COVID-19), and work increasingly needs to be performed through ICTs [1]. Whereas the ubiquity of ICT software and devices has made it possible for employees to work from anywhere or at any time, it has also made it common for employees to stay connected during non-working hours [2]. For instance, employees commonly deal with work emails, participate in online conferences, and respond to work-related messages on platforms after office hours. The use of ICTs blurs the boundaries between work and non-work, increasing employee work complaints [3] and unethical behavior [4]. These challenges have also raised concerns among managers and researchers about off-hours work connectivity [5]. Unfortunately, there is no consensus on the impact of the work connectivity behavior after-hours. Contrary to the conclusion that there is a positive impact on work connectivity behavior after-hours [6], European countries, such as Portugal and Belgium, and companies, such as BMW and Volkswagen, have prohibited work connectivity during non-working hours on the grounds that this behavior harms the physical and mental health of their employees [7]. Therefore, given the divergence between theory and practice regarding work connectivity behavior after-hours, it is necessary to re-examine its impact on employees.

Although previous studies have explored the impact of work connectivity behavior after-hours on employees’ psychological state at work and performance, there is a lack of consensus on these conclusions for several reasons. First, the positive or negative effects of work connectivity behavior after-hours are generally studied separately. There is no doubt that research on the positive aspect of work connectivity behavior after-hours expands its benefits [8], and research on its negative aspect amplifies its harm to employees [9,10]. This suggests that discussing the positive and negative aspects of work connectivity behavior after-hours separately is the potential reason for the lack of consensus on the conclusions of work connectivity behavior after-hours. Furthermore, although a previous study by Ren et al. (2023) [11] examined the influence of work connectivity behavior after-hours in terms of both positive and negative aspects, their study of job performance neglected to consider its impact on employees’ psychological state at work [12]. This limits our understanding of how work connectivity behavior after-hours affects employees’ psychological state at work. Lastly, the existing conclusions on work connectivity behavior after-hours have primarily been derived from research in formal workplaces [13], which ignores the unique context where employees are compelled to work remotely or from home. This lack of attention to the specific context of working from home has resulted in biased perceptions of the relationship between work connectivity behavior after-hours and employee outcomes. Given the increasing prevalence of work connectivity behavior after-hours in organizations [1], understanding how and when it influences employees’ psychological state at work is crucial not only for organizational functioning but also for resolving the connectivity paradox.

To address the aforementioned issues, this study explores the consensus on the relationship between work connectivity behavior after-hours and employees’ psychological state at work through work engagement, and narrows the gap between the theory and practice of work connectivity behavior after-hours. This study is based on the buffering hypothesis of the Job Demands–Resources (JD-R) model and the context of employees having to work from home, which reveals the positive impact of work connectivity behavior after-hours on work engagement through increased work autonomy and reduced emotional exhaustion. This finding provides a new explanatory perspective for the integration of inconsistent conclusions about work connectivity behavior after-hours. In addition, by recognizing the differences in the influence of work connectivity behavior after-hours between managers and ordinary employees, the potential reasons for the contradiction between the theory and practice of work connectivity behavior after-hours were revealed. Ultimately, our conclusions have implications regarding whether organizations should allow work connectivity behavior after-hours and how to manage it effectively.

## 2. Theoretical Background

The JD-R model is widely used to explain the impact of work resources and demands on employee work-related outcomes. Work resources and personal resources are seen as supportive factors that help employees complete their tasks, reduce psychological stress, and promote personal growth [14,15]. Work demands refer to the tasks, pressures, and responsibilities that employees need to deal with in their work [14]. The JD-R model consists of three core assumptions: (1) the “double pathway” hypothesis, which posits that work resources and demands have both beneficial and detrimental effects on employees [16]; (2) the “buffer” hypothesis, which suggests that work resources can buffer the negative impact of a high level of job demands on employees and mitigate their negative effects [15,17], especially when work resources are continuously available; and (3) the “coping” hypothesis, which proposes that employees are more likely to convert work resources into high levels of work performance in a challenging environment [18].

According to the buffering hypothesis of the JD-R model, work resources not only promote the positive performance of employees but also buffer the negative effects of work demands on employees [19,20]. It has been proven that work connectivity behavior after-hours is a resource for employees to complete their work when working from home. On the one hand, work connectivity behavior after-hours provides employees with work resources such as autonomy and control during remote work [21], which helps employees choose a suitable time to engage in work, thereby enhancing work continuity and engagement. On the other hand, work connectivity behavior after-hours can alleviate the influence of family interference on work, reducing work–family conflict and emotional exhaustion during remote work [8], and thereby reducing the negative impact on work engagement. Therefore, based on the buffering hypothesis of the JD-R model, we constructed and tested a theoretical model to examine the impact of work connectivity behavior after-hours on work engagement. The results show that work connectivity behavior after-hours can positively impact work engagement by increasing employees’ work autonomy and reducing emotional exhaustion.

In addition, we further explore the conditions under which work connectivity behavior after-hours has a positive impact on work engagement. Whereas work connectivity behavior after-hours can provide employees with resources such as work autonomy, previous studies have indicated that employees with different workplace statuses have varying needs for work resources [22,23]. Especially in contexts where employees are required to work remotely, managers have greater work responsibilities, face more complex tasks, and face higher communication demands; thus, they have a higher need for work autonomy [24,25]. In contrast, ordinary employees have relatively simpler job tasks and a lower need for work autonomy, but may be more concerned about self-resource depletion during remote work [26]. This indicates that, during remote work, managers are more focused on the improvement in work efficiency brought about by work connectivity behavior after-hours [27], while ordinary employees are more concerned about alleviating the emotional exhaustion caused by work connectivity behavior after-hours [10]. Therefore, we explored the difference in the impact of work connectivity behavior after-hours on work engagement from the perspective of workplace status to better understand the underlying reasons for the discrepancy between theory and practice regarding work connectivity behavior after-hours.

## 3. Hypothesis Development

### 3.1. Work Connectivity Behavior After-Hours and Work Engagement

Work connectivity behavior after-hours refers to the use of ICTs or devices by employees to engage in work activities or contact colleagues during non-work hours [28]. This is a work behavior that employees adopt to complete their work tasks during non-working time [29]. Its crucial role is to enable employees to accomplish work tasks through ICTs during non-working hours [28]. This behavior leads to the permeation of work into employees’ non-work domains and has a significant impact on their work and personal lives. Existing studies have shown that work connectivity behavior after-hours can increase employees’ control over work and promote their creativity by providing employees with opportunities to work flexibly [30]. Meanwhile, previous studies have found that work connectivity behavior after-hours contributes to an increase in employees’ role-integration capabilities, which, in turn, affects their psychological state at work [31]. For example, the research conducted by Van Zoonen et al. (2023) [29] showed that work connectivity behavior after-hours can help alleviate employee exhaustion. It can be seen that work connectivity behavior after-hours not only improves the performance of employees but also promotes a positive psychological state at work (e.g., engagement and satisfaction).

Work engagement, such as a positive psychological state at work, plays a crucial role in employee performance. Schaufeli et al. (2002) [32] defined work engagement as a positive, fulfilling, work-related psychological state characterized by vigor, dedication, and absorption. Consistent with the argument of the JD-R model, numerous studies have demonstrated that working environment and work forms are important factors influencing the work-related psychological states of employees [33], and different work forms can influence the level of work engagement by altering job demands and resources for employees [34,35]. Therefore, we posit that remote work will alter the impact of work connectivity behavior after-hours on employees’ work engagement. This is because work connectivity behavior after-hours has been validated in existing studies to create a positive work environment that promotes work engagement by enabling employees to flexibly arrange their work schedules and enhancing their sense of control [7]. Furthermore, communication and emotional connections with the organization are strengthened when employees work from home, improving their enthusiasm for and engagement in work [8]. These roles, to some extent, mitigate the negative effects on their work engagement that may arise when employees feel a lack of care and connection with the organization [36]. In summary, we suggest that work connectivity behavior after-hours has a positive effect on employees’ work engagement, and thus propose the following hypothesis.

**H_1_:**  *Work connectivity behavior after-hours will be positively related with employee work engagement during remote work*.

### 3.2. The Mediating Role of Work Autonomy

Work autonomy refers to the right of employees to make free and independent decisions regarding their work progress and planning, which is the embodiment of employees’ independent control of their work [37,38]. Employees can attain more positive psychological states at work in conditions with work autonomy, such as having a strong sense of responsibility [37], enhancing their perceived self-effectiveness, creativity, and proactivity [39,40]. Previous studies have shown that employees perceive greater work autonomy in non-fixed and flexible work arrangements [35,41]. The study conducted by Cavazotte et al. (2014) [10] demonstrated that work connectivity behavior after-hours breaks the boundaries of work time and space, and enhances the freedom and capacity for self-decision in employees’ work. Additionally, Sherling and Shirom (2005) [42] indicated that work connectivity behavior after-hours can improve the efficiency of communication and feedback between employees and the organization, increase the opportunities for employee involvement in decision-making processes, and enhance the degree of autonomy in arranging work. Therefore, we argue that work connectivity behavior after-hours without fixed schedules will increase employees’ work autonomy, as it provides flexible time options, freedom, and independent decisions regarding how employees accomplish their work tasks.

Moreover, work autonomy contributes to employees’ positive work-related psychological states and behaviors while working from home [43]. The JD-R model suggests that job resources have natural motivational properties that motivate and increase work engagement [17]. Firstly, existing studies have indicated that work autonomy can enable employees to experience higher self-efficacy and perceive more meaningful work, which, in turn, fosters a sense of work responsibility and increases their work engagement [44]. Secondly, recent studies have shown that work autonomy allows for employees to choose an appropriate timing for their work, reducing interference and interruptions from family, enhancing their work focus, facilitating a coherent and immersive work experience, and ultimately increasing their work engagement [45,46]. These studies highlight the significance of work autonomy as a critical job resource for increasing employee work engagement. Therefore, we posit that work connectivity behavior after-hours can increase employees’ work engagement by increasing work autonomy, and thus propose the following hypothesis.

**H_2_:**  *Work autonomy will play a mediating role in the relationship between work connectivity behavior after-hours and employees’ work engagement*.

### 3.3. The Mediating Role of Emotional Exhaustion

The JD-R model argues that the presence of work autonomy does not exclude the explanatory mechanism of emotional exhaustion [18]. Research on the JD-R model has indicated that job demands and resources can have both positive and negative effects on employees’ work-related psychological states [47]. Therefore, we suggest that work connectivity behavior after-hours not only affects employees’ work autonomy but also impacts their emotional exhaustion. Emotional exhaustion is a psychological fatigue caused by the feeling of being physically and mentally exhausted, as a result of prolonged exposure to work-related pressures [48]. Evidence indicates that employees experiencing emotional exhaustion lack sufficient resources and energy to devote to work, resulting in negative outcomes such as a lack of concentration and work burnout [49,50], with a detrimental impact on work engagement.

Previous studies have shown that work stress, frustration, and work–family conflict are important factors leading to employees’ emotional exhaustion [51], while forms of work that alleviate work stress and work–family conflict can help reduce employees’ emotional exhaustion. Research on work connectivity behavior after-hours indicates that it increases employees’ work resources, such as work autonomy and flexibility [52], which not only helps to alleviate work stress but also mitigates the need for conflict between work and family, reducing employees’ emotional resource depletion [53]. In addition, work connectivity behavior after-hours strengthens the connection between employees and organizations during remote work, enhances employees’ sense of organizational identification and belongingness, and satisfies employees’ social interaction needs [30], thus improving employees’ positive emotional resources. Therefore, according to the “buffer” hypothesis of the JD-R model, we posit that work connectivity behavior after-hours can reduce the negative impact on employees’ work engagement by reducing emotional exhaustion, and thus propose the following hypothesis.

**H_3_:**  *Emotional exhaustion will play a mediating role in the relationship between work connectivity behavior after-hours and employees’ work engagement*.

### 3.4. The Moderating Role of Workplace Status

Although we believe that work autonomy and emotional exhaustion are mechanisms that explain the relationship between work connectivity behavior after-hours and work engagement, the effects of work autonomy and emotional exhaustion will be influenced by contextual factors such as workplace status [54]. Workplace status refers to the perceived prestige and respect from others in the workplace that employees experience [23], which can be categorized into managers and ordinary employees [55]. Previous research has shown that workplace status influences employees’ cognition, behavior, and emotions [56], and that employees with different roles (manager vs. ordinary employee) will exhibit different expectations regarding flexible work arrangements [22]. First, employees with different workplace statuses have different work contents. Managers often need to manage multiple tasks that cross organizational boundaries simultaneously [25], which demands a higher level of work autonomy that can be provided by work connectivity behavior after-hours [57]. In contrast, ordinary employees have relatively simpler tasks and less demand for work autonomy. They are more inclined to use work connectivity behavior after-hours to alleviate work and emotional pressures [20]. Second, workplace status will influence employees’ attitudes toward work connectivity behavior after-hours due to differences in work contents [58]. Managers will take advantage of the autonomy in work provided by work connectivity behavior after-hours to engage in more positive work behaviors and ensure task completion [24]. However, ordinary employees often find themselves in a passive state of connectivity and may have difficulties perceiving the work autonomy provided by work connectivity after-hours [59]. Finally, workplace status will affect employees’ access to work resources. Managers have access to more resources to manage work stress and emotional exhaustion, making them more likely to utilize the work resources provided by work connectivity behavior after-hours to complete more work [60]. Ordinary employees have relatively weak access to work resources [61], and thus tend to prioritize the use of limited work resources to alleviate work and emotional pressures. Therefore, we argue that workplace status will influence the mechanism through which work connectivity behavior after-hours affects work engagement, and consider the workplace status as a boundary condition that affects the relationship between work connectivity behavior after-hours and work engagement; thus, we propose the following hypothesis.

**H4:**  *Workplace status will affect the positive impact mechanism of work connectivity behavior after-hours on work engagement; managers will explain the relationship between work connectivity behavior after-hours and work engagement through work autonomy, and ordinary employees will explain it through emotional exhaustion*.

**Figure 1:**
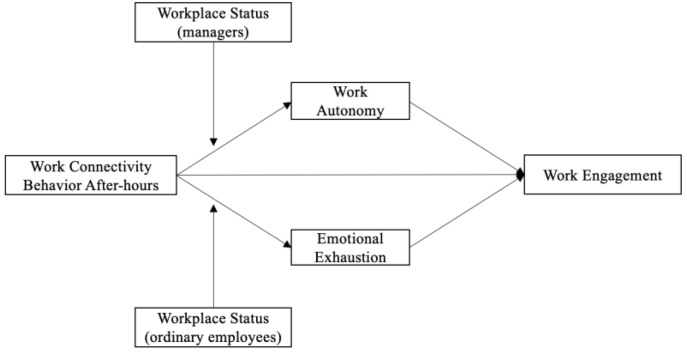
The theoretical model.

Based on the above views, we propose a theoretical model of this study, as shown in Figure 1.

## 4. Research Methods

### 4.1. Participants and Procedure

We collected data through the online survey platform Credamo (Beijing, China). Participants were selected based on their full-time employment and experience of working from home during the COVID-19 pandemic. The survey was conducted in two waves to reduce common method bias. We also provided CNY 3 to participants who completed questionnaires and met our quality criteria, respectively. The first wave (4 October 2020) measured participants’ basic information, such as gender, age, and position, as well as their work connectivity behavior after-hours and work autonomy. The second wave (24 October 2020) measured participants’ information related to the number of children and work–family conflict, as well as emotional exhaustion and work engagement. Finally, a total of 376 questionnaires were collected for our study, and 257 of them were valid, with an effective response rate of 68.4%. To ensure the validity of the questionnaires, detection items and reverse items were included in both waves of the survey to assess participants’ work experience and response sincerity. In subsequent analyses, the reverse-coded items were re-coded. 

The basic characteristics of the sample are as follows. In terms of gender, 135 participants were female, accounting for 53%, and 122 were male, accounting for 47%. In terms of age, 126 participants were aged 18–30, accounting for 49%; 113 (44%) were aged 31–40; 17 (6.6%) were aged 41–50; only 1 was aged 51–60, accounting for 0.4%. In terms of education, 3 (1.2%) participants had a high school education or less; 20 (7.8%) had specialist education, accounting for 7.8%; 206 had a bachelor’s degree, accounting for 80.2%; 28 had a master’s degree or above, accounting for 10.9%. In terms of positions, 85 participants were ordinary employees, accounting for 33%, and 172 were managers, accounting for 67%. In terms of parenting, 36 participants did not raise children, accounting for 14%; 185 raised one child, accounting for 72%; 36 raised two or more children, accounting for 14%.

### 4.2. Measures

To ensure accurate and effective measurement of the research constructs, our study combined the research context and the target participants, utilizing established measurement scales that have been widely used in previous research. These scales were translated into Chinese using a back-translation procedure following the guidelines of Schaffer and Riordan (2003) [62]. The questionnaire was scored on a 5-point Likert scale, with 1 indicating “strongly disagree” and 5 indicating “strongly agree”, unless otherwise specified.

Work connectivity behavior after-hours (WCA) was measured through 6 items from Fenner and Renn (2010) [63], such as “I use my phone at home in the evenings or on weekends to complete work tasks that were not finished during the day”. The Cronbach’s α value in this study was 0.906. Considering the study context of working from home, employees are likely to face both demands for after-hours work from their colleagues as well as the need to voluntarily engage in work during off-hours in order to achieve a family–work balance. To better understand the impact of work connectivity behavior after-hours on employees’ psychological state at work, we chose the scale conducted by Fenner and Renn for a measurement of work connectivity behavior after-hours. This is because Fenner and Renn’s scale focuses more on how employees connect to work during non-work hours.

Work autonomy (WA) was measured through 3 items from Liu et al. (2007) [64], such as “I decide how to do my work”. The Cronbach’s α value in this study was 0.737.

Emotional exhaustion (EE) was measured through 3 items from Watkins et al. (2015) [65], such as “Just the thought of facing another workday makes me feel exhausted”. The Cronbach’s α value in this study was 0.910.

Work engagement (WE) was measured through 9 items from Schaufeli et al. (2002) [32], such as “At work, I feel bursting with energy”. The Cronbach’s α value in this study was 0.917.

Workplace status (WS) was categorized into managers and ordinary employees based on the perspective of managerial responsibilities, as classified by Martin et al. (2006) [55].

Control variables: previous research has shown that individual differences can lead to different perceptions of work connectivity behavior after-hours and individuals exhibit different emotional states and work attitudes. In addition, during the work-from-home period of the COVID-19 pandemic, family and the COVID-19 infection could have also affected employees’ emotions and psychological state at work. To mitigate the impact of these factors on our results, we controlled for factors such as gender (Gender), age (Age), education level (Edu), number of children (Child), work–family conflict (WFC), and the number of COVID-19 infections in the local area (Infection). The number of COVID-19 infections was obtained from the daily COVID-19 reports published by the provincial health commission, selecting the cumulative confirmed cases as of 28 October 2020. Work–family conflict was measured through 9 items from Carlson et al. (2000) [66], such as “My job often keeps me from participating in family activities”. The Cronbach’s α value in this study was 0.917.

## 5. Results

The analytical tools AMOS 22.0, SPSS 21.0, and PROCESS V3.3 macro were used in this study. Specifically, we used the descriptive statistics function of SPSS to analyze the mean and standard deviation of the variables, the correlate function of SPSS 21.0 to analyze the correlations of key variables, and the dimension reduction function of SPSS 21.0 to test for common method bias. AMOS 22.0 was used to test the fit of the theoretical model and the common method bias. The research hypothesis was tested using the regression function of SPSS, and the mediation effect was verified by PROCESS V3.3 macro.

The results are presented in two parts: descriptive statistics and hypothesis testing. The mean, standard deviation, correlation, common method bias, and theoretical model fit of key variables are presented in the descriptive statistics part. In the hypothesis testing part, the hypothesis was tested in terms of the total sample, the manager sample, and the ordinary employee sample. Firstly, a regression analysis was carried out on the total sample followed by an analysis of the ordinary employee sample, and finally an analysis of the manager sample. Hypotheses H1, H2, and H3 were verified by analysis of the total sample, and hypothesis H4 was verified by analysis of the sample of ordinary employees and managers.

### 5.1. Descriptive Statistics

We tested the correlation relationships between variables, mean of core variables, and common method bias (CMB) using correlation and factor analysis by SPSS 21.0. The results (Table 1) showed that the mean (standard deviation) values of work connectivity behavior after-hours, work autonomy, emotional exhaustion, and work engagement were 3.98 (0.80), 4.08 (0.58), 2.32 (1.07), and 3.80 (0.73), respectively. Work connectivity behavior after-hours was significantly positively correlated with work autonomy (r = 0.196, *p* < 0.01) and work engagement (r = 0.141, *p* < 0.05), and significantly negatively correlated with emotional exhaustion (r = −0.152, *p* < 0.05). Work autonomy was significantly positively correlated with work engagement (r = 0.450, *p* < 0.01) and significantly negatively correlated with emotional exhaustion (r = −0.269, *p* < 0.01). Emotional exhaustion was significantly negatively correlated with work engagement (r = −0.485, *p* < 0.01). We used Harman’s method, using SPSS 21.0 to test for common method bias. The results showed that the first factor in the four-factor model accounted for 35.7% of the variance, which is less than 50%. These results indicate that there was no serious common method bias in the single-source data used in our study [67].

In addition, we also conducted a confirmatory factor analysis (CFA) using Amos 22.0 to examine the model fit among work connectivity behavior after-hours, work autonomy, emotional exhaustion, and work engagement. The results indicated that the four-factor model was significantly better than the single-factor model and the best models with two or three factors (Table 2).

### 5.2. Hypothesis Testing

**Figure 2:**
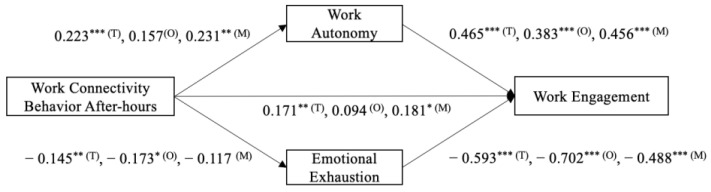
The analysis results. Note. *** *p* < 0.001, ** *p* < 0.01, * *p* < 0.05. T = results of total sample, O = results of ordinary employee sample, M = results of manager sample.

Our study used hierarchical regression analysis and the PROCESS macro in SPSS 21.0 to test the hypothesis (Figure 2). In addition, we used the Bootstrap method to examine the mediation and moderation effects involved in the study [68]. The results can be found in Table 3. M0, M2, and M4 represent the regression results of the control variables on work autonomy, emotional exhaustion, and work engagement, respectively. M1 shows the regression result of work connectivity behavior after-hours on work autonomy, indicating a significant positive effect of work connectivity behavior after-hours on work autonomy (β = 0.223, *p* < 0.001). M3 presents the regression result of work connectivity behavior after-hours on emotional exhaustion, demonstrating a significant negative effect of work connectivity behavior after-hours on emotional exhaustion (β = −0.145, *p* < 0.01). M5 displays the regression result of work connectivity behavior after-hours on work engagement, indicating a significant positive effect of work connectivity behavior after-hours on work engagement (β = 0.171, *p* < 0.01); thus, H1 was supported. Combining M1 and M6, it can be observed that work autonomy played a fully mediating role in the relationship between work connectivity behavior after-hours and work engagement (indirect effect = 0.09, 95% CI= [0.03, 0.16]); thus, H2 was supported. Combining M3 and M7, it can be seen that emotional exhaustion played a fully mediating role in the relationship between work connectivity behavior after-hours and work engagement (indirect effect = 0.07, 95% CI= [0.02, 0.14]); thus, H3 was supported.

Further, Table 4 shows the regression analysis results of the ordinary employees. M0a, 2a, and 4a represent the regression results of the control variables on work autonomy, emotional exhaustion, and work engagement, respectively. M1a and M3a represent the regression results of work connectivity behavior after-hours on work autonomy and emotional exhaustion. The results indicated that work connectivity behavior after-hours did not have a significant effect on work autonomy among ordinary employees (r = 0.157, *p* > 0.05), but had a significant negative effect on emotional exhaustion (r = −0.173, *p* < 0.05). M5a showed that work connectivity behavior after-hours did not have a significant effect on work engagement among ordinary employees (r = 0.094, *p* > 0.05). Combining M1a and M6a, it can be observed that work autonomy did not mediate the relationship between work connectivity behavior after-hours and work engagement (indirect effect = 0.05, 95% confidence interval [−0.01, 0.15]). Combining M3a and M7a, it can be seen that emotional exhaustion played a fully mediating role in the relationship between work connectivity behavior after-hours and work engagement (indirect effect = 0.11, 95% CI= [0.01, 0.26]).

The regression results obtained in the analysis of managers can be seen in Table 5. M0b, 2b, and 4b represent the regression results for the control variables on work autonomy, emotional exhaustion, and work engagement, respectively. M1b and M3b represent the regression results of work connectivity behavior after-hours on work autonomy and emotional exhaustion. The results indicated that work connectivity behavior after-hours had a significant positive effect on work autonomy among managers (r = 0.231, *p* < 0.01), but did not have a significant effect on emotional exhaustion (r = −0.117, *p* > 0.05). M5b showed that work connectivity behavior after-hours had a significant positive effect on work engagement among managers (r = 0.181, *p* < 0.05). Combining M1b and M6b, it can be observed that work autonomy played a fully mediating role in the relationship between work connectivity behavior after-hours and work engagement (indirect effect = 0.09, 95% CI= [0.03, 0.18]). Combining M3b and M7b, it can be seen that emotional exhaustion did not mediate the relationship between work connectivity behavior after-hours and work engagement (indirect effect = 0.04, 95% CI= [−0.003, 0.123]).

This analysis indicated that workplace status is a boundary condition for the impact of work connectivity behavior after-hours on work engagement. For ordinary employees, work connectivity behavior after-hours can positively affect work engagement by decreasing emotional exhaustion, but cannot increase work engagement through increased work autonomy. For managers, work connectivity behavior after-hours can increase work engagement by increasing work autonomy, but cannot affect work engagement through reduced emotional exhaustion; thus, H_4_ was supported.

## 6. Discussion

The results of this study demonstrate that work connectivity behavior after-hours has a significant positive impact on employees’ work engagement (β = 0.171, *p* < 0.01). This is different from the previous negative conclusion that work connectivity behavior after-hours reduced work engagement [69,70]. Work connectivity behavior after-hours can provide employees with more work resources to help them complete tasks, such as flexibility [41], especially when employees working from home face work–family conflicts and work interruptions from family members. Therefore, work connectivity behavior after-hours will be seen by employees working from home as a work resource for mitigating interruptions and achieving progress toward work goals rather than a work demand, which, in turn, increases employee engagement in work connectivity behavior after-hours.

Further, the results of this study indicate that work autonomy and emotional exhaustion are important mechanisms to explain how work connectivity behavior after-hours affects employee work engagement. This is different from the emotional exhaustion that has often been used to explain the negative impact of work connectivity behavior after-hours on employees’ work outcomes [71]. However, in the context of working from home, we found that work autonomy and emotional exhaustion can simultaneously contribute to the positive effect of work connectivity behavior after-hours on work engagement; work connectivity behavior after-hours can have a positive impact on work engagement by increasing work autonomy and reducing emotional exhaustion. According to the buffer assumption of the JD-R model, we believe that work connectivity behavior after-hours helps employees working from home to balance their work–home life and provides them with the necessary freedom to complete their work tasks, thus buffering the loss of emotional resources caused by work connectivity behavior after-hours. Therefore, work connectivity behavior after-hours can increase employee work engagement by enhancing work autonomy and reducing emotional exhaustion.

In addition, the results of this study demonstrate that the impact of work connectivity behavior after-hours on work engagement differs among employees with different workplace statuses. Considering the perspective of workplace status, we found that, in managers with a high workplace status, work connectivity behavior after-hours only had a positive effect on work engagement by increasing work autonomy, but in ordinary employees with a low workplace status, work connectivity behavior after-hours only increased the positive impact on work engagement by reducing emotional exhaustion. This is because employees with different workplace statuses have different perceptions of and needs for work connectivity behavior after-hours [55]. Especially in the context of working from home, managers perceive work connectivity behavior after-hours as a tool for task allocation and coordination, while ordinary employees view it as a resource for achieving progress in work goals, increasing communications with the organization, and reducing emotional exhaustion. Therefore, the impact of work connectivity behavior after-hours on managers’ work engagement will be achieved through increased work autonomy, while the impact on ordinary employees’ work engagement will be achieved through reduced emotional exhaustion.

## 7. Theoretical Contributions

We have made several theoretical contributions in the following aspects. First, this study reveals the positive impact and mechanisms of work connectivity behavior after-hours on work engagement based on the JD-R model, which provides a new explanatory perspective for understanding the inconsistent conclusions of work connectivity behavior after-hours. Previous studies have examined the effects of work connectivity behavior after-hours on work–family balance [72], work performance [11], and employee psychological disengagement [29], etc. These studies suggested that work connectivity behavior after-hours may have both positive and negative effects [8], leading to divergent conclusions on work connectivity behavior after-hours. Meanwhile, these studies did not explore how work connectivity behavior after-hours affects employees’ psychological state at work. In the context of working from home, we examined the effects of work connectivity behavior after-hours on work engagement and the role of work autonomy and emotional exhaustion, showing that work connectivity behavior after-hours has a positive impact on work engagement among employees working from home and a positive impact on work engagement by increasing work autonomy and reducing emotional exhaustion. This finding not only expands the literature on work connectivity behavior after-hours from the perspective of work engagement, but also contributes to a consensus on the role of work connectivity behavior after-hours.

Second, this study clarifies the differences in the impact of work connectivity behavior after-hours on the work engagement of managers and ordinary employees from the perspective of workplace status, and the results reveal the potential reasons for the inconsistencies in the conclusions of research on work connectivity behavior after-hours. Although discussing the boundary role of work–family segmentation preferences [72], psychological entitlement and the organizational support climate [73] in work connectivity behavior after-hours is important for understanding work connectivity behavior after-hours, it is not sufficient to explain the inconsistent conclusions regarding work connectivity behavior after-hours. In the context of working from home, our study finds that the differential perceptions of and needs for work connectivity behavior after-hours between managers and ordinary employees, caused by differences in workplace status, are the reasons for this contradiction. Work connectivity behavior after-hours only increases the work autonomy of managers and only reduces the emotional exhaustion of ordinary employees. This finding not only enriches the understanding of the boundary conditions of work connectivity behavior after-hours but also reveals the importance of workplace status in influencing employees’ perceptions and work behaviors, further highlighting the significance of workplace status in organizational behavior research.

Third, this study expands on the role of the JD-R model of work connectivity behavior after-hours and the literature on the buffering hypothesis of the JD-R model. Existing research has focused excessively on the “dual pathway” hypothesis of the JD-R model and has extensively discussed the positive or negative effects of work characteristics such as work connectivity behavior after-hours [29,74]. However, the neglect of the buffering hypothesis of the JD-R model has led to a biased understanding of work connectivity behavior after-hours and has also limited our understanding of the JD-R model. Our study on work-from-home employees reveals the role of the buffering hypothesis of the JD-R model in the relationship between work connectivity behavior after-hours and work engagement. We also found that workplace status was a boundary condition for the application of the JD-R model. This finding further highlights the important role of the buffering hypothesis of the JD-R model in explaining the relationship between work characteristics and work outcomes, and reveals that workplace status is an important boundary condition for the JD-R model.

## 8. Practical Implications

Our work also has several practical implications, as follows. First, organizations need to clarify the scope of the use of work connectivity behavior after-hours. In contrast to previous research findings [9], our study of employees working from home shows that work connectivity behavior after-hours can increase employee work engagement. This suggests that work connectivity behavior after-hours has different effects in different work situations. Therefore, organizations need to rationalize the use of work connectivity behavior after-hours according to their actual work contexts and leverage the positive impact of work connectivity behavior after-hours in different environments, such as working from home, remote work, and flexible working. Second, organizations need to emphasize the work resource aspect of work connectivity behavior after-hours and provide employees with more autonomy in their work. Our study found that work connectivity behavior after-hours served as a work resource that increased work autonomy and reduced emotional exhaustion, thereby positively impacting work engagement. Therefore, organizations should fully leverage the resource aspect of work connectivity behavior after-hours and avoid it becoming a work demand. This can be achieved by adjusting organizational work practices and performance assessments based on the needs of remote work and flexible work scenarios [1]. Finally, organizations need to pay attention to the differences in the perception of management practices among employees with different workplace statuses. Our analysis of managers and ordinary employees revealed differences in the impact of work connectivity behavior after-hours, whereby it only increased managers’ work autonomy, and reduced emotional exhaustion for ordinary employees. It can be seen that managers and ordinary employees have different needs regarding work connectivity behavior after-hours. Therefore, organizations should not only adopt targeted measures for work connectivity behavior after-hours to address the diverse needs of different employees, but should also consider the differences and needs of various employees when forming other policies to enhance the effectiveness of management’s measures.

## 9. Limitation

The present study has several limitations that should be addressed and improved in future research. First, the data collection in this study relied on self-reported measures from employees in China, and all core variables were measured using five-point scales in questionnaires. This reliance on subjective employee perceptions inevitably introduces biases in the research findings. At the same time, the sample that included only Chinese employees ignored the potential impact of different cultures on the results. In future research, objective data, such as connectivity frequency, duration, the frequency and degree of employee interruptions, and samples from different cultures should be utilized to analyze the impact of off-hours connectivity. Second, due to the limitations of our data, this study was carried out during the COVID-19 pandemic, which limited its ability to reveal the dynamic processes through which work connectivity behavior after-hours influences work engagement. Future research should adopt longitudinal research designs to explore the dynamic effects of off-hours connectivity on employees’ work and personal lives in a normal work and life context. Third, this study analyzed the effects of work connectivity behavior after-hours on work engagement separately from the perspectives of managers and ordinary employees, without conducting a matched analyses between the two groups, which limited the ability to further understand the differences between the initiators and recipients of connectivity. Future research will shed light on the impact of work connectivity behavior after-hours from the perspectives of connectivity initiators and recipients. Finally, this study only focuses on the impact of work connectivity behavior after-hours on work engagement, ignoring the consideration of employees’ health and well-being. This limitation limits the understanding of the impact of work connectivity behavior after-hours. Future research will more broadly explore the effects of work connectivity behavior after-hours on work–family balance, occupational health, well-being, and work behavior.

In addition to the abovementioned limitations, we believe that our research provides interesting directions for future research regarding the following aspects, and future research can provide more valuable insights into work connectivity behavior after-hours by addressing these aspects. First, given the positive impact of work connectivity behavior after-hours on work engagement when working from home, we suggest that future studies should explore its positive effects in other domains (e.g., the family domain). With the popularity of remote and hybrid models, exploration of the family domain is urgent and necessary. Second, given the significance of workplace status in explaining different mechanisms, we suggest that future research should further explore employees’ work behavior based on differences in workplace status. This would contribute to enhancing our understanding of the needs and behaviors of managers and ordinary employees at work.

## 10. Conclusions

In conclusion, this study, using the JD-R model, revealed the positive effects of work connectivity behavior after-hours on employees’ work engagement and its underlying mechanisms. These findings indicate that, in the context of working from home, work connectivity behavior after-hours can have a positive impact on work engagement by increasing managers’ work autonomy and reducing emotional exhaustion for ordinary employees. Our study advances the understanding of the positive effects of work connectivity behavior after-hours and provides a potential explanation for the current inconsistent conclusions about work connectivity behavior after-hours from the perspective of workplace status. In addition, our study demonstrates the role of the JD-R model in explaining the positive effects of work connectivity behavior after-hours and expands the buffer hypothesis of the JD-R model. It also offers insights for organizations managing work connectivity behavior after-hours in remote work or flexible work models.

## Figures and Tables

**Table 1:** Results for variable correlation, mean, and S.D.

Variables	Gender	Age	Edu	Infection	Child	WFC	WCA	WA	EE	WE
Gender	1									
Age	0.084	1								
Edu	0.017	−0.028	1							
Infection	0.048	−0.148 *	−0.041	1						
Child	0.071	0.211 **	0.087	0.000	1					
WFC	−0.009	−0.182 **	0.059	0.039	−0.109	1				
WCA	0.045	0.056	0.123 *	0.013	0.067	0.183 **	1			
WA	−0.004	0.118	−0.007	−0.014	0.043	−0.275 **	0.166 **	1		
EE	0.054	−0.159 *	0.028	0.071	−0.148 *	0.602 **	−0.033	−0.404 **	1	
WE	−0.026	0.085	0.093	−0.039	0.194 **	−0.301 **	0.129 *	0.527 **	−0.579 **	1
Mean	0.470	1.580	3.010	3945.91	1.010	2.783	3.984	4.084	2.316	3.802
S.D.	0.500	0.633	0.484	14,246.202	0.552	0.950	0.791	0.577	1.065	0.728

Note. ** *p* < 0.01, * *p* < 0.05. WFC is work–family conflict, WCA is work connectivity behavior after-hours, WA is work autonomy, EE is emotional exhaustion, WE is work engagement, S.D. is Standard Deviation.

**Table 2:** Results of confirmatory factor analysis.

Model	χ2	df	χ2/df	CFI	TLI	RMSEA	SRMR
One-factor model	1740.579	189	9.209	0.529	0.477	0.179	0.171
Two-factor model	823.575	188	4.381	0.807	0.784	0.115	0.078
Three-factor model	507.99	186	2.731	0.902	0.890	0.082	0.063
Four-factor model	419.921	183	2.295	0.928	0.917	0.071	0.053

Note. One-factor model: WCA + WA + EE + WE; two-factor model: WCA, WA + EE + WE; three-factor model: WCA, EE, WA + WE; four-factor model: WCA, WA, EE, WE.

**Table 3:** Results of regression analysis (total).

Model	M0	M1	M2	M3	M4	M5	M6	M7
Variables	WA	WA	EE	EE	WE	WE	WE	WE
Gender	−0.013	−0.020	0.066	0.071	−0.041	−0.047	−0.037	−0.004
Age	0.073	0.054	−0.035	−0.023	0.002	−0.012	−0.037	−0.026
Edu	0.011	−0.013	0.000	0.016	0.096	0.078	0.084	0.087
Infection	0.008	0.004	0.040	0.043	−0.022	−0.025	−0.027	0.000
Child	−0.001	−0.014	−0.082	−0.073	0.157 *	0.147 *	0.153 **	0.103 *
WFC	−0.263 ***	−0.307 ***	0.586 ***	0.615 ***	−0.289 ***	−0.322 ***	−0.180 **	0.042
WCA		0.223 ***		−0.145 **		0.171 **	0.068	0.086
WA							0.465 ***	
EE								−0.593 ***
R^2^	0.081	0.128	0.377	0.397	0.128	0.156	0.344	0.368
ΔR^2^	0.081	0.047	0.377	0.020	0.128	0.028	0.216	0.240
F	3.669 ***	5.206 ***	25.251 ***	23.429 ***	6.135 ***	6.575 ***	16.273 ***	18.032 ***
N	257	257	257	257	257	257	257	257

Note. *** *p* < 0.001, ** *p* < 0.01, * *p* < 0.05, WFC is work–family conflict, WCA is work connectivity behavior after-hours, WA is work autonomy, EE is emotional exhaustion, WE is work engagement.

**Table 4:** Results of regression analysis (ordinary employees).

Model	M0a	M1a	M2a	M3a	M4a	M5a	M6a	M7a
Variables	WA	WA	EE	EE	WE	WE	WE	WE
Gender	0.109	0.067	0.030	0.077	0.038	0.013	−0.013	0.067
Age	0.163	0.143	−0.017	0.005	0.026	0.014	−0.041	0.018
Edu	0.002	0.001	0.059	0.060	−0.091	−0.092	−0.092	−0.050
Infection	−0.031	−0.035	0.102	0.107	0.014	0.012	0.025	0.087
Child	−0.180	−0.181	−0.121	−0.120	0.100	0.099	0.168	0.015
WFC	−0.451 ***	−0.496 ***	0.612 ***	0.661 ***	−0.458 ***	−0.485 ***	−0.295 *	−0.021
WCA		0.157		−0.173 *		0.094	0.034	−0.027
WA							0.383 ***	
EE								−0.702 ***
R^2^	0.251	0.271	0.467	0.492	0.273	0.281	0.388	0.531
ΔR^2^	0.251	0.020	0.467	0.025	0.273	0.008	0.115	0.258
F	4.350 ***	4.099 ***	11.378 ***	10.652 ***	4.887 ***	4.293 ***	6.012 ***	10.750 ***
N	85	85	85	85	85	85	85	85

Note. *** *p* < 0.001, * *p* < 0.05, WFC is work–family conflict, WCA is work connectivity behavior after-hours, WA is work autonomy, EE is emotional exhaustion, WE is work engagement.

**Table 5:** Results of regression analysis (managers).

Model	M0b	M1b	M2b	M3b	M4b	M5b	M6b	M7b
Variables	WA	WA	EE	EE	WE	WE	WE	WE
Gender	−0.072	−0.050	0.105	0.094	−0.104	−0.087	−0.064	−0.041
Age	−0.066	−0.066	0.013	0.014	−0.120	−0.121	−0.091	−0.114
Edu	−0.056	−0.081	0.008	0.021	0.146 *	0.126	0.163	0.136 *
Infection	−0.013	−0.006	0.034	0.031	−0.081	−0.077	−0.074	−0.062
Child	−0.008	−0.013	0.005	0.007	0.067	0.063	0.069	0.067
WFC	−0.184 *	−0.224 **	0.572 ***	0.592 ***	−0.205 **	−0.236 **	−0.134 **	0.053
WCA		0.231 **		−0.117		0.181 *	0.075	0.124
WA							0.456 ***	
EE								−0.488 ***
R^2^	0.044	0.095	0.340	0.353	0.095	0.125	0.314	0.279
ΔR^2^	0.044	0.051	0.340	0.013	0.095	0.030	0.219	0.184
F	1.269	2.447 ***	14.185 ***	12.795 ***	2.870 ***	3.359 ***	9.329 ***	7.896 ***
N	172	172	172	172	172	172	172	172

Note. *** *p* < 0.001, ** *p* < 0.01, * *p* < 0.05, WFC is work–family conflict, WCA is Work connectivity behavior after-hours, WA is Work autonomy, EE is Emotional exhaustion, WE is Work engagement.

## Data Availability

The raw data supporting the conclusions of this article will be made available by the authors, without undue reservation.
